# Methyl-Sensitive Amplification Polymorphism (MSAP) Analysis Provides Insights into the DNA Methylation Changes Underlying Adaptation to Low Temperature of *Brassica rapa* L.

**DOI:** 10.3390/plants13131748

**Published:** 2024-06-24

**Authors:** Lijun Liu, Wanpeng Wang, Xiaoming Lu, Tianyu Zhang, Junyan Wu, Yan Fang, Li Ma, Yuanyuan Pu, Gang Yang, Wangtian Wang, Wancang Sun

**Affiliations:** 1 State Key Laboratory of Aridland Crop Science, College of Agronomy, Gansu Agricultural University, Lanzhou 730070, China; liulj198910@163.com (L.L.); lxm955991@163.com (X.L.); 15352107840@163.com (T.Z.); ffyv@163.com (Y.F.); mal@gsau.edu.cn (L.M.); vampirepyy@126.com (Y.P.); yangg@gsau.edu.cn (G.Y.); wtwang@gsau.edu.cn (W.W.); 2 Zhangye Academy of Agricultural Sciences, Zhangye 734000, China; 17609364826@163.com

**Keywords:** *Brassica rapa* L., low temperature, MSAP, DNA methylation, gene expression

## Abstract

Background: DNA methylation can change rapidly to regulate the expression of stress-responsive genes. Previous studies have shown that there are significant differences in the cold resistance of winter rapeseed (*Brassica rapa* L.) after being domesticated in different selection environments; however, little is known about the epigenetic regulatory mechanisms of its cold resistance formation. Methods: Four winter rapeseed materials (‘CT-2360’, ‘MXW-1’, ‘2018-FJT’, and ‘DT-7’) domesticated in different environments were selected to analyze the DNA methylation level and pattern changes under low temperature using methylation-sensitive amplified polymorphism technology with 60 primer pairs. Results: A total of 18 pairs of primers with good polymorphism were screened, and 1426 clear bands were amplified, with 594 methylation sites, accounting for 41.65% of the total amplified bands. The total methylation ratios of the four materials were reduced after low-temperature treatment, in which the DNA methylation level of ‘CT-2360’ was higher than that of the other three materials; the analysis of methylation patterns revealed that the degree of demethylation was higher than that of methylation in ‘MXW-1’, ‘2018-FJT’, and ‘DT-7’, which were 22.99%, 19.77%, and 24.35%, respectively, and that the methylation events in ‘CT-2360’ were predominantly dominant at 22.95%. Fifty-three polymorphic methylated DNA fragments were randomly selected and further analyzed, and twenty-nine of the cloned fragments were homologous to genes with known functions. The candidate genes *VQ22* and *LOC103871127* verified the existence of different expressive patterns before and after low-temperature treatment. Conclusions: Our work implies the critical role of DNA methylation in the formation of cold resistance in winter rapeseed. These results provide a comprehensive insight into the adaptation epigenetic regulatory mechanism of *Brassica rapa* L. to low temperature, and the identified differentially methylated genes can also be used as important genetic resources for the multilateral breeding of winter-resistant varieties.

## 1. Introduction

The production of rapeseed in recent years has been grim as the fifth major crop after rice, wheat, maize, and soybean. Rapeseed is also the most important oil crop; thus, the cultivation of rapeseed has significant economic value for the oil crop industry in China. The climate in the northern region of China is harsh and cold and the growing conditions are poor; therefore, traditional spring rapeseed varieties do not adapt well to the harsh natural conditions. However, winter rapeseed is a good winter/spring cover crop and the only over-wintering oilseed crop in the northern region; its planting can not only alleviate the shortage of usable oil but also reduce the wind erosion of the soil and improve the ecological environment [1]. Winter rapeseed is sown in autumn and returns to green in the following spring, with an overwintering period of up to half a year. The primary challenge for winter rapeseed production is to improve cold resistance to ensure successfully overwintering, so resolving the cold resistance mechanism of winter rapeseed is an important scientific issue [2]. Previous studies have reported that winter rapeseed can overwinter in the north area of 35° N due to the effect of alternating dry and cold environments on improving the cold tolerance of varieties and the formation of a wide range of adaptations [3,4,5]. Current research on the cold resistance mechanism of winter rapeseed has been insufficient to resolve the adaptive mechanism to different breeding environments; therefore, analyzing the formation mechanism of cold resistance in a strong cold-resistant variety is of great meaning for the genetic improvement of winter rapeseed.

As sessile organisms, plants have evolved a series of complex strategies (e.g., transcriptional modulation, epigenetic regulation, and physiological and metabolic rearrangements) to regulate growth and development in response to the environment to improve survivability, and thus to adapt to different growth environments during long-term adaptive processes. During the adaptation of maize from tropical to temperate climate regions, the products associated with multiple metabolic pathways changed significantly due to environmental selection [6]. Soybean is a temperate crop that has a natural variation at the J locus that can improve its adaptation to the tropics [7]. Previous studies have revealed that DNA methylation, as an adaptive mechanism, changes rapidly in specific or global regions to regulate the expression of stress genes when plants are subjected to stressful environmental stimuli and are involved in a variety of biological processes, such as plant growth and development, stress response (including cold, heat, drought, salt, etc.) and genomic evolution, which is often used to instruct long-term bioprocesses (e.g., the evolution of genomes) and short-term procedures (e.g., responding to external pressures) [8,9,10]. DNA methylation provides a novel origin of variation, and its potential to increase stress adaptability brings new research orientations for breeders in enhancing the resistance of crops by serving as a relatively stable, heritable, and trans-generational epigenetic marker [8]. Reversible dynamic changes in DNA methylation and demethylation regulate gene expression, enabling plants to evolve favorable phenotypes. Natural selection acts on these epigenetic variations to dynamically regulate epigenetic alleles for the formation of adaptations that promote plant evolution [11,12]. Temperature-related epitope alleles exhibit species-specific methylation patterns during the adaptation of heteropolyploid orchids [13]. Such differential methylation patterns can also occur in different ecotypes of the same species with phenotypic differences due to environmental changes. For example, tropical and temperate sacred lotus ecotypes (*Nelumbo nucifera*) are adapted to low and high latitudes, respectively, and exhibit distinct methylome patterns [14]. Thus, epimutation is an important evolutionary force in plants, and mutant phenotypes obtained through methylation changes can respond rapidly to natural selection. In addition, methylated DNA regions have high mutation rates and may be the first step toward a more stable sequence mutation [15]. In conclusion, environmental stressors cause specific adaptations and evolutionary traits by influencing methylation levels and patterns. More in-depth studies on the functions and mechanisms of DNA methylation in a wider variety of species will help us gain a more comprehensive understanding of the regulatory mechanisms of DNA methylation in plant adaptation. In recent years, significant progress has been made in understanding the regulatory mechanisms of DNA methylation involved in the stress acclimation of *Arabidopsis thaliana* [16], *Rice* [17], *Gossypium hirsutum* [18], and *Brassica napus* L. [19], but the mechanism of DNA methylation in the stress adaption of *Brassica rapa* L. remains unclear.

With the advancement of DNA methylation research and the development of detection technologies, many techniques have been used to detect changes in DNA methylation, including methylated DNA immunoprecipitation (MeDIP), methyl-CpG binding domain-based capture (MBDCap) [20], methylation-sensitive amplified polymorphism (MSAP) [21,22], and whole-genome bisulfite sequencing (WGBS) [23]. Depending on sequencing resolution and cost, affinity enrichment-based methods (MEDIP and MBDCap) are suitable for rapid, large-scale, and low-resolution studies, restriction endonuclease-based methods (MSAP) for site-specific/targeting studies, and bisulfite transformation-based methods (WGBS) for high-resolution studies [9].

Our previous research showed that winter rapeseed Longyou-7 could improve cold resistance after domestication in different cultivation regions with widely varying temperatures, and the physiological/biochemical characteristics and the overall methylation levels of DNA (HPLC analysis) of the different materials changed significantly after treatment with the DNA methylation inhibitor 5-azaC under low temperature [5]. In the present study, four materials, including ‘CT-2360’ (Cao Tan, Dingxi), ‘MXW-1’ (Shangchuan, Lanzhou), ‘2018-FJT’ (Fanjiatun, Jilin), and ‘DT-7’ (Datong, Shanxi), which were domesticated in different environments with different cold resistance, were used to analyze the changes in DNA methylation levels and patterns at 4 °C using MSAP technology with 60 primer pairs. The polymorphic primers were screened, and the differentially methylated DNA bands were recovered and cloned and their sequences were compared. Then, the differentially methylated genes were selected for real-time fluorescence quantitative polymerase chain reaction (PCR) analysis to provide a theoretical basis for further analyzing the DNA methylation regulatory mechanism of the formation of cold resistance in winter rapeseed Longyou-7. This approach will provide important candidate genes for the creation of cold-resistant winter rapeseed germplasms.

## 2. Results

### 2.1. Pre-Amplification Reactions and Screening of Polymorphic Primers

The EcoRI/HpaII (E+H) and EcoRI/MspI (E+M) double-digested products were connected to specific adapters, and the ligation products were used as templates for the PCR reaction using the pre-amplification primers E-pre and HM-pre (Supplementary Table S1). The PCR products were detected via gel electrophoresis, and the diffuse distribution of the bands indicated successful digestion and ligation (Figure S1). Then, the 60 primer pairs were used for selective amplification in the MSAP experiment. All bands obtained following polyacrylamide gel electrophoresis were counted, and 18 pairs of polymorphic primers were screened according to the number of amplified bands, the clarity of the bands, and high polymorphism (Supplementary Table S2).

### 2.2. Effect of Low Temperature on DNA Methylation Levels in Winter Rapeseed

**Figure 1:**
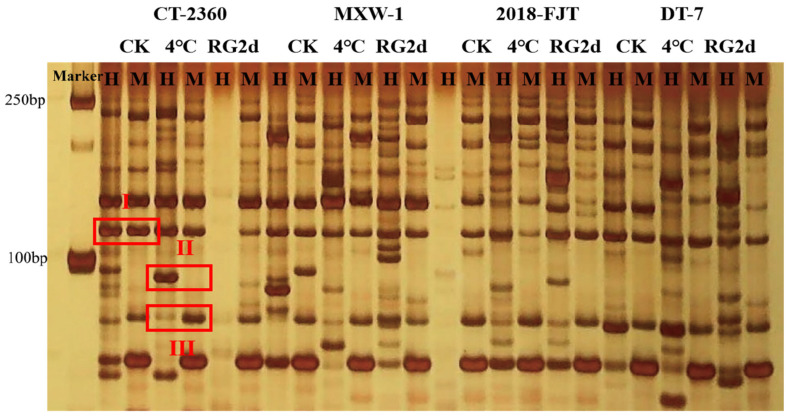
MSAP electrophoresis map of winter rapeseed under low temperature.

The bands obtained following polyacrylamide gel electrophoresis were classified into type I, type II, and type III, in which band type I indicated that both E+H and E+M had bands, suggesting that the ‘CG’ site was not methylated; band type II: E+H with band and E+M without band indicates hemi-methylation of the ‘CG’ site; and band type III: E+H with no band and E+M with a band, i.e., the ‘CG’ site was holomethylated (Figure 1). A total of 1426 clear 100–250 bp bands were amplified in ‘CT-2360’, ‘MXW-1’, ‘2018-FJT’, and ‘DT-7’ at low temperature, of which 243 hemi-methylated sites accounted for 17.04% of the total amplified bands, 351 holomethylated sites accounted for 24.61% of the total amplified bands, and 594 methylated sites accounted for 41.65% of the total amplified bands (Table 1). The number of hemi-methylated sites of ‘CT-2360’, ‘MXW-1’, ‘2018-FJT’, and ‘DT-7’ at room temperature was 37, 28, 35, and 35, accounting for 20.56%, 15.22%, 19.13%, and 18.82% of all sites, respectively; the number of holo-methylated sites was 51, 48, 42, and 39, accounting for 28.33%, 26.09%, 22.95%, and 20.97% of all sites, respectively. The number of semi-methylated sites of ‘CT-2360’, ‘MXW-1’, ‘2018-FJT’, and ‘DT-7’ was 33, 29, 20, and 26 after 24 h of low temperature at 4 °C, which represented 19.88%, 15.51%, 11.98%, and 15.03% of all sites, respectively; and the number of holo-methylated sites was 47, 41, 46, and 37, which occupied the total amplified bands of 28.31%, 21.93%, 27.54%, and 21.39%, respectively.

There were 35, 23, 41, and 27 hemi-methylated sites after 2 d of recovery at 24 °C in ‘CT-2360’, ‘MXW-1’, ‘2018-FJT’, and ‘DT-7’, comprising 20.59%, 13.07%, 22.28%, and 15.17% of all sites, respectively, and 48, 39, 47, and 59 holo-methylated sites, making up 28.24%, 22.16%, 25.54%, and 33.15% of the total number of amplified bands, respectively. In summary, the hemi-methylation ratios were always less than the holo-methylation ratios at room temperature and low temperature in the four winter rapeseed materials selected from the different environments, and from the results of the holo-methylation ratios and total methylation ratios, the methylation level of ‘CT-2360’ was the highest, and the total methylation ratios of ‘MXW-1’, ‘2018-FJT’, and ‘DT-7’ decreased under low-temperature stress, suggesting that significant demethylation occurred. Nevertheless, the total methylation ratios of ‘2018-FJT’ and ‘DT-7’ increased significantly after 2 d of recovery at 24 °C.

### 2.3. Effect of Low Temperature on DNA Methylation Patterns in Winter Rapeseed

**Figure 2:**
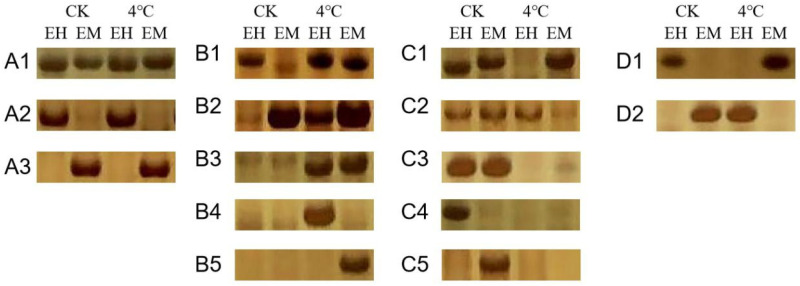
DNA methylation pattern of *Brassica rapa* L. EH: EcoRI/HpaII double digestion; EM: EcoRI/MspI double digestion.

Changes in the DNA methylation patterns of winter rapeseed selected from four different environments after low-temperature stress at 4 °C were classified into four categories (Figure 2). Category A was a monomorphic band, with no change in methylation at the same band before and after low-temperature treatment. This methylation pattern accounted for the highest percentage, and ‘CT-2360’ had the largest proportion at 62.30% in the four materials. In contrast, categories B, C, and D were polymorphic bands. Bands of category B represented the degree of DNA methylation that decreased after low temperature, i.e., demethylation occurred, with ‘MXW-1’, ‘2018-FJT’, and ‘DT-7’ more demethylated by 22.99%, 19.77%, and 24.35%, respectively, and ‘CT-2360’ being the least demethylated at 14.75%. Category C indicated the methylation/hypermethylation pattern under low temperature, with ‘CT-2360’ showing the highest degree of methylation at 22.95%. In addition, the bands in category D were indistinguishable from methylation or demethylation and accounted for a smaller percentage of the bands (Table 2). These results indicate that the overall trend of DNA methylation in ‘MXW-1’, ‘2018-FJT’, and ‘DT-7’ was demethylation during low-temperature stress, while ‘CT-2360’ was mainly methylated.

### 2.4. Effect of Room Temperature Recovery (RG2d) on DNA Methylation Patterns in Winter Rapeseed

Changes in the DNA methylation patterns of the four materials selected from different environments after RG2d are shown in Table 3. Consistent with the results at 4 °C, the highest proportion of unmethylated patterns of the four materials was in type A but accounted for less than the pattern under low temperature. The degree of demethylation (type B) in ‘MXW-1’, ‘2018-FJT’, and ‘DT-7’ was higher at 19.02%, 22.1%, and 21.72%, respectively, and demethylation of ‘CT-2360’ was the lowest at 13.55%. The methylation pattern (type C) of the four materials was higher after RG2d than those in the low-temperature treatment, with ‘CT-2360’ having the largest proportion (36.13%), and ‘MXW-1’, ‘2018-FJT’, and ‘DT-7’ having methylation patterns of 22.7%, 26.16%, and 27.78%, respectively.

### 2.5. Sequence Analysis of Differentially Methylated DNA Fragments

A group of 64 polymorphic DNA methylation bands screened from the MSAP experiment with 18 primer pairs were cloned, purified, and sequenced. The BLAST X analysis in the NCBI database revealed that 53 sequences were successfully matched, and the average size of the cloned bands was 112 bp, ranging from 53 to 214 bp (Table 4). Of these, 29 fragments had high homology to genes with known functions, including anti-freeze proteins, such as endo-1,3-beta-glucosidase, VQ motif-containing protein 22 (*VQ22*), cysteine-rich receptor-like protein kinase 10 (*CRK10*), protein phosphatase 2C (*PP2C*), auxin-induced protein 15A (*AUX15A*, *LOC103834602*), and glycine-rich RNA-binding proteins 5 (*GR-RBP5*, *LOC103860185*) that improve stress tolerance. In addition, one fragment was homologous to endonuclease 8 which is necessary for cellulose formation in the cell wall and glucosyltransferases that are involved in the accumulation of anthocyanin, respectively. Meanwhile, some fragments were involved in the synthesis of secondary metabolites, including the E3 ubiquitin-protein ligase *ATL6-like*, terpenoid synthase 17, and the cytoplasmic male sterility-associated cytochrome oxidase subunit I (*coxI-2*) gene. In contrast, 14 sequences were highly homologous to uncharacterized proteins. Moreover, no genetic information was obtained from the other 10 cloned fragments, but chromosomal information as well as chloroplast and mitochondrial gene sequences were included, suggesting that (de)methylation events also occur in the genome. These results demonstrate that genes regulated by cold-induced (de)methylation may be involved in a variety of cellular functions.

### 2.6. Cloning and Expression Profiling of the Polymorphic Fragments

**Figure 3:**
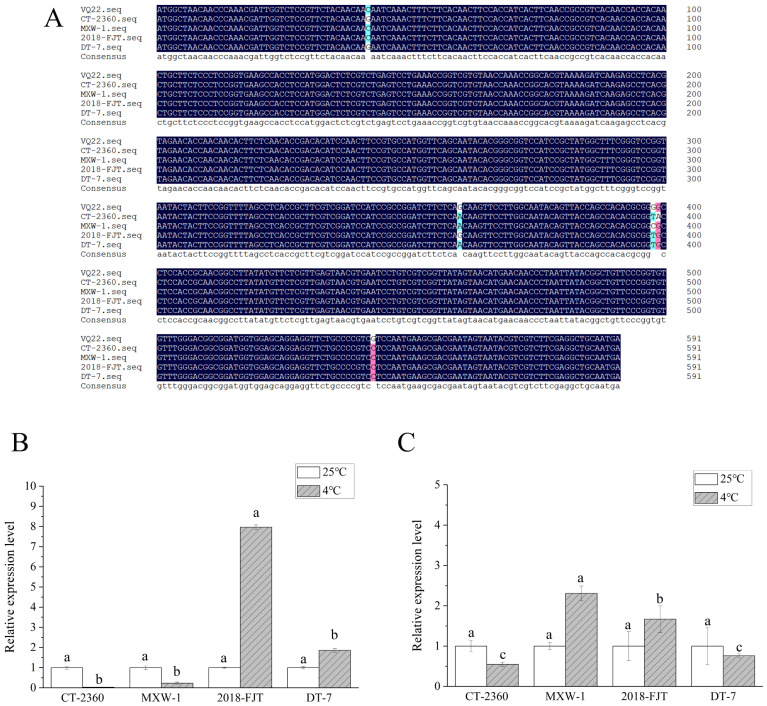
Multiple alignment and relative expression level of *VQ22* in the four winter rapeseed materials: (**A**) multiple alignment of *VQ22* sequence in the four materials; (**B**) relative expression level of the *VQ22* conservative sequence under low temperature conditions; (**C**), relative expression level of the *VQ22*-specific sequence under low temperature conditions. Different letters indicate significant difference at *p* < 0.05 level among the different materials.

**Figure 4:**
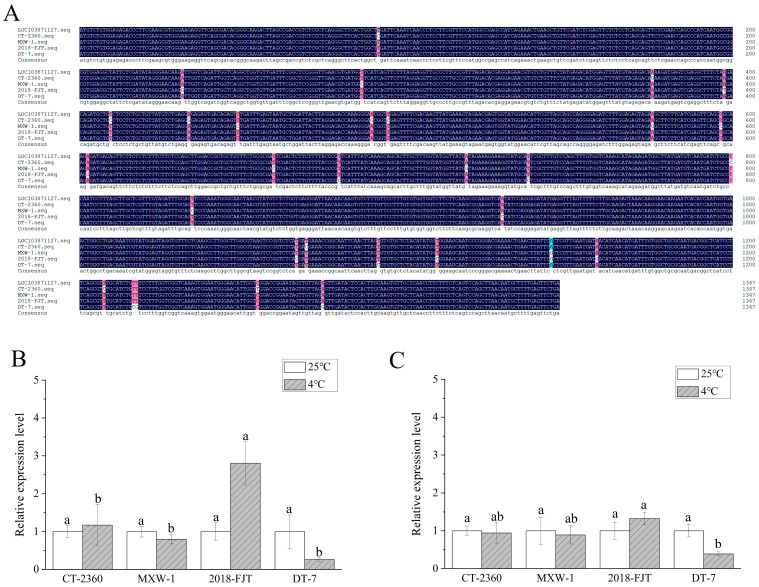
Multiple alignment and relative expression level of *LOC103871127* in the four winter rapeseed materials: (**A**) multiple alignment of the *LOC103871127* sequence in the four materials; (**B**) relative expression level of the *LOC103871127* conservative sequence under low temperature; (**C**) relative expression level of the *LOC103871127*-specific sequence under low temperature. Different letters indicate significant difference at the *p* < 0.05 level among the different materials.

To verify the pattern of the methylation changes under low temperature, *VQ22* and *LOC103871127* were selected to clone and measure the expression levels in the four materials via qPCR at 25 °C and 4 °C. As shown in Figure 3, the expression levels of *VQ22* conserved and specific sequences in ‘MXW-1’, ‘2018-FJT’, and ‘DT-7’ were higher than that in ‘CT-2360’ under low temperature. In the conserved region, the relative expression of *VQ22* was upregulated in ‘CT-2360’ and ‘MXW-1’ and downregulated in ‘2018-FJT’ and ‘DT-7’, with the highest expression in ‘2018-FJT’ and the lowest expression in ‘CT-2360’. In the specific region, *VQ22* was upregulated in the strongly cold-resistant materials ‘MXW-1’ and ‘2018-FJT’ but downregulated in ‘CT-2360’ and ‘DT-7’. As shown in Figure 4, the expression level of *LOC103871127* showed the same trend in conserved and specific sequences. It was upregulated in the strongly cold-resistant material ‘2018-FJT’, and downregulated in ‘DT-7’, whereas its expression was essentially unchanged in ‘CT-2360’ and ‘MXW-1’. In some cases, the expression levels of these two genes were consistent with changes in methylation status detected using MSAP. For instance, the expression of *VQ22* was downregulated in the less cold-resistant material ‘CT-2360’ due to a methylation event, whereas *VQ22* and the uncharacterized gene *LOC103871127* underwent demethylation in the strongly cold-resistant material ‘2018-FJT’ resulting in upregulated expression. These results show that the expression of these two selected genes changed significantly under low temperature, suggesting that *VQ22* and *LOC103871127* may play important roles in the adaptation of winter rapeseed to low temperature.

## 3. Discussion

It is well known that environmental stress is a major factor affecting the yield of field crops. Plants have evolved a series of strategies for responding to the ambient environment, including transcriptional modulation [24,25], metabolic rearrangements [25,26,27], and epigenetic regulation [28,29,30]. As one of the earliest discovered and most studied regulatory mechanisms in epigenetics, DNA methylation/demethylation is involved in a variety of biological processes, such as plant growth and development [31], stress response [9,32,33], and genome evolution [10,34], and can be used as a plant adaptive mechanism [35]. Winter rapeseed, as the only overwintering cruciferous oilseed crop in the Northwest of China, must tolerate a severe growing environment with extremely low temperatures [36,37]; thus, it is of great significance to study the cold resistance of winter rapeseed. Previous studies have investigated morphological structures, physiological and biochemical characteristics [38], and molecular mechanisms using transcriptome [2,39], proteome [40,41], and small RNA sequencing [42] of cold stress on *Brassica rapa* L.; however, few studies focus on its intricate adaptive mechanism. It has been shown that winter rapeseed can be domesticated in different cultivation regions with large temperature differences to improve its cold tolerance [3,4,5], and the physiological/biochemical characteristics and overall DNA methylation levels of the different materials change significantly after treatment with the DNA methylation inhibitor 5-azaC under low-temperature conditions [5], but little is known about the epigenetic regulation of its adaptive mechanism to different breeding environments. Several techniques have been used to detect changes in DNA methylation; MSAP is a reliable and cost-effective technique for site-specific/targeting studies [9]. Therefore, in the present study, using the MSAP technique, we found a pronounced difference in the DNA methylation levels and patterns of the four materials that differed in cold resistance after domestication. In addition, 64 polymorphic fragments were screened and further cloned, purified, and sequenced. Of these, 29 fragments were involved in biological processes, such as the abiotic stress response and the synthesis of secondary metabolites. Furthermore, mRNA expression of the differentially methylated genes *VQ22* and *LOC103871127* was tested via qPCR analysis. This study provides an in-depth understanding of the effect of DNA methylation on the regulatory mechanisms of winter rapeseed adaptation and provides crucial genetic resources for the multilateral breeding of winter-resistant varieties.

In general, DNA cytosine methylation is an important epigenetic modification that affects gene expression during various adaptive processes and has been analyzed in a variety of plants using the MSAP technique. For example, the DNA methylation level is significantly higher during rice anthesis in a susceptible rice group, and significantly lower in a tolerant rice group under high-temperature conditions [43]. In *Lolium perenne* [44], the total methylation level decreases after exposure to drought. Strong cold-resistant varieties of *Brassica napus* L. are predominantly demethylated under low temperature conditions; however, weaker cold-resistant materials are predominantly methylated [45]. It is generally believed that plants coordinate the expression of stress-responsive genes through the reversible reactions of DNA methylation and demethylation. Cold stress in *Zea mays* L. leads to changes in the extent and pattern of DNA methylation, with demethylation predominating [46]. In *Chorispora bungeana*, alterations in DNA methylation levels and patterns have also been observed across periods of chilling and freezing [47]. Additionally, MSAP analyses of *Vicia villos* growing in high-altitude (Xingyi) and low-altitude (Dafang) regions of Guizhou revealed that the total methylation and hemi-methylation rates of *V. villos* from the low-altitude region were lower than those from the high-altitude Xingyi region, suggesting that DNA undergoes a higher degree of demethylation in the *V. villos* inhabiting the lower altitude region [48], which agrees with the results of the present experiments. In this study, the DNA methylation levels of ‘CT-2360’, ‘MXW-1’, ‘2018-FJT’, and ‘DT-7’ decreased after 24 h of low-temperature treatment, resulting in a demethylation event (Figure 1, Table 1). The hemi-methylation and holomethylation rates of ‘2018-FJT’ and ‘DT-7’ selected from the Fanjiatun, Jilin and Datong, and Shanxi regions, which are at low altitude, were significantly lower than those of ‘CT-2360’ from the high-altitude region. The DNA methylation level of ‘CT-2360’ decreased by 1.43% after low-temperature stress compared with that at room temperature, whereas the DNA methylation levels of the strongly cold-resistant materials ‘MXW-1’, ‘2018-FJT’, and ‘DT-7’ decreased by 9.37%, 6.08%, and 8.45%, respectively, compared with those at room temperature. The decrease in the DNA methylation level at low temperature was greater in the strongly cold-resistant materials than in the less cold-resistant material. Meanwhile, DNA methylation/hypermethylation and DNA demethylation patterns coexisted in the four winter rapeseed materials (Figure 2, Table 2), in which ‘MXW-1’, ‘2018-FJT’, and ‘DT-7’ were more demethylated under low-temperature conditions, and ‘CT-2360’ was methylated/hypermethylated to a higher extent. These results indicate that DNA methylation and demethylation events both occurred at 4 °C (low temperature) in the four domesticated materials, consistent with the findings by Liu et al. [49] in *Brassica rapa* in that cold acclimation induces both the up- and downregulation of methylation. Furthermore, the DNA methylation levels of all four materials increased after recovery of growth at room temperature (Table 1 and Table 3). Thus, we speculate that DNA methylation plays an important role in the low-temperature adaptation of *Brassica rapa* L.

It is well established that DNA methylation is associated with the silencing of genes, while demethylation turns on gene expression [50,51]. Zhu et al. [52] investigated anthocyanin enrichment in peaches during low-temperature storage and found that the higher transcript levels of anthocyanin synthase-related genes were associated with reduced methylation levels in their promoter regions. The promoter of the *ACS1* and *ETR1* genes in *Arabidopsis* hyperhydricity involved in the ethylene pathway displays CHH demethylation patterns, which consequently causes the upregulation of these two genes and increases ethylene enrichment [53]. In the current study, random sequencing of the cold-induced polymorphic DNA methylation bands revealed that some of these genes were highly homologous with the anti-freeze proteins endo-1,3-beta-glucosidase, VQ motif-containing protein 22 (*VQ22*), cysteine-rich receptor-like protein kinase 10 (*CRK10*), protein phosphatase 2C (*PP2C*), auxin-induced protein 15A (*AUX15A*), and glycine-rich RNA-binding protein 5 (*GR-RBP5*), of which the difference was most pronounced for *VQ22* (Table 4). Among these annotated genes, *CRK10* belongs to the RD subclass of Ser/Thr kinases, which participates in the control of stress responses and development [54]. *PP2C* and *AUX15A* are involved in the ABA signaling pathway [55], and *GR-RBPs* are vital post-transcriptional RNA metabolism regulator factors in crops during abiotic environmental stresses [56]. *VQ22* is widely observed in living organisms, plays an important role in plant growth, development, and stress tolerance, and is induced by cold [57,58,59]. The qPCR analysis results showed that in the less cold-resistant material ‘CT-2360’, the expression of *VQ22* was downregulated due to a methylation event, whereas demethylation of *VQ22* occurred in the strongly cold-resistant material ‘2018-FJT’, resulting in upregulated expression (Figure 3). In addition to the annotated genes, a number of uncharacterized proteins also altered the methylation status, of which *LOC103871127* was demethylated in the strongly cold-resistant material ‘2018-FJT’, resulting in upregulated expression (Figure 4). However, the effect of these genes on the cold tolerance of winter rapeseed is unknown. Further investigation of DNA methylation involved in the expression of these genes under low-temperature stress will be interesting in the future.

## 4. Materials and Methods

### 4.1. Plant Materials and Cold Treatment

Of the four materials selected for this experiment, ‘MXW-1’, ‘2018-FJT’, and ‘DT-7’ were strongly cold-resistant materials, and ‘CT-2360’ was a weakly cold-resistant material. Among them, ‘MXW-1’ (original designation of Longyou-7) was selected in Shangchuan, Lanzhou, with ‘CT-2360’ as the parent, as it can overwinter safely in Raohe (Heilongjiang), Gongzhuling (Jilin), and Altay (Xinjiang). ‘DT-7’ and ‘2018-FJT’ were new lines obtained from ‘MXW-1’ after 4 years of selection in Datong (Shanxi) and Fanjiatun (Gongzhuling, Jilin) (Table 5) [5]. Seedlings grown to the five-leaf stage were treated in a low-temperature chamber (16 h light and 8 h dark) at 4 °C for 24 h followed by 24 °C recovery growth for 2 d (RG2d). Each treatment contained three biological replicates. The harvested leaves were immersed immediately in liquid nitrogen and stored at −80 °C for further DNA and RNA extraction. 

### 4.2. DNA Extraction, RNA Extraction, and cDNA Synthesis

Genomic DNA was extracted using the CTAB method [60] and purified with chloroform-isoamyl alcohol. Then, the quality and concentration of the DNA were determined with an ultra-micro nucleic acid/protein analyzer (No. DS-11+; DeNovix, Wilmington, DE, USA). Total RNA extraction and reverse transcription were performed according to the instructions with the plant RNA extraction (No. DP452; Tiangen, Beijing, China) and reverse transcription kits (No. RR036A; TaKaRa, Dalian, China).

### 4.3. MSAP Analysis

Sixty primer pairs were selected for the MSAP experiment to analyze the levels and patterns of genomic DNA methylation in the four materials domesticated from different environments at 4 °C for 24 h with recovery at 24 °C for 2 d. The adapters, pre-amplified primers, and selected primers used in the experiment were obtained from previous studies [4,61] (Supplementary Table S1). The three endonucleases used in the experiment, such as EcoRI, HpaII, and MspI, were purchased from NEB (R3101V, R0170V, and R0106VR); EcoRI/HpaII and EcoRI/MspI were used for double enzyme cleavage. Based on the results, the experimental conditions were continuously optimized to select the appropriate digestion time and pre-amplification dilution; the different bands were counted, methylation levels were calculated, and the methylation patterns were analyzed.

### 4.4. Different Bands Were Recovered and Cloned and the Sequences Were Compared

According to the method of Du et al. [62], the polymorphic target bands on the polyacrylamide gel were recovered, and the recovered supernatant (1 µL) was used as the template for amplification with selected amplification primers. The PCR products were detected via 1% agarose gel electrophoresis. A single target product of the same size was recovered according to the instruction manual with the agarose gel DNA recovery kit (No. AG21005; Accurate Biotech, Changsha, Hunan). The product was ligated with the pMD^TM^19-T vector (No. D102A; TaKaRa, Dalian, China) and transformed into Transl-T1 competent cells, and appropriate positive colonies were selected for sequencing. The sequencing results were searched for homologous and sequence comparisons using the BLAST tool on the NCBI website (http://www.ncbi.nlm.nih.gov/ (accessed on 29 November 2020)).

### 4.5. qPCR Analysis of the Differentially Methylated Genes

After cloning the CDS sequence of *VQ22* and *LOC103871127*, DNAMAN software was used for the multiple alignment of *VQ22* and *LOC103871127* among the four materials. Then, the primers were designed for qPCR analysis according to the conserved and specific regions (Supplementary Table S3). qPCR amplification was performed on a QuantStudio 5 real-time PCR system (ABI, Foster City, CA, USA) with SYBR Green I (Takara, RR820A) to verify the MSAP results. The relative expression levels of the genes to the reference gene *β-actin* were determined using the 2^−ΔΔCt^ method [63]. Three independent technical replicates and three biological replicates were performed for each gene in a single sample, and the mean and standard error were calculated.

## 5. Conclusions

This study provides a preliminary understanding of the differences in methylation levels and patterns in four winter rapeseed varieties selected from different environments under low-temperature treatment. The main results highlight that the variation in DNA methylation level and pattern may play an important role in the adaptation of winter rapeseed to low temperatures. Moreover, several differentially methylated genes, such as *VQ22* and *LOC103871127*, have been suggested to regulate low-temperature tolerance via DNA cytosine methylation in different ways. We believe that DNA methylation/demethylation is an essential epigenetic mechanism for the adaption of *Brassica rapa* L. to different breeding environments with fluctuating temperatures. The identified candidate genes are valuable genetic resources for in-depth research on the epigenetic regulatory mechanisms of winter rapeseed adaptation. We hope that our research will open a new direction to accelerate the genetic improvement of super-cold-resistant winter rapeseed varieties.

## Figures and Tables

**Table 1:** Changes in DNA methylation level in *Brassica rapa* L. subjected to the low temperature (4 °C for 24 h) and 24 °C recovery growth for 2 d (RG2d). Band type I: unmethylated; Band type II: semi-methylation; Band type III: hypermethylation; total number of bands = Band type I + Band type II + Band type III; total number of methylated bands = Band type II + Band type III; semi-methylation ratio = Band type II/total number of bands; holomethylation ratio = Band type III/total number of bands; total methylation ratio = total number of methylated bands/total number of bands.

Materials	Treatment	Band Type I: The Number of Unmethylated Bands	Band Type II: The Number of Semi-Methylated Bands	Band Type III: The Number of Total Methylated Bands	Total Number of Bands	Total Number of Methylated Bands	Semi- Methylation Ratio (%)	Holo-methylation Ratio (%)	Total Methylation Ratio (%)
CT-2360	CK	92	37	51	180	88	20.56	28.33	48.89
	4 °C	86	33	47	166	80	19.88	28.31	48.19
	RG2d	87	35	48	170	83	20.59	28.24	48.82
MXW-1	CK	108	28	48	184	76	15.22	26.09	41.30
	4 °C	117	29	41	187	70	15.51	21.93	37.43
	RG2d	114	23	39	176	62	13.07	22.16	35.23
2018-FJT	CK	106	35	42	183	77	19.13	22.95	42.08
	4 °C	101	20	46	167	66	11.98	27.54	39.52
	RG2d	96	41	47	184	88	22.28	25.54	47.83
DT-7	CK	112	35	39	186	74	18.82	20.97	39.78
	4 °C	110	26	37	173	63	15.03	21.39	36.42
	RG2d	92	27	59	178	86	15.17	33.15	48.31
Total		1221	369	544	2134	913			

**Table 2:** Effects of low temperature on the DNA methylation pattern in *Brassica rapa* L. The different types are shown in Figure 2. EH and EM represent the results of EcoRI/HpaII and EcoRI/MspI double digestion, respectively. The presence (1)/absence (0) were determined based on the polyacrylamide gel electropherograms of 18 polymorphic primer pairs during control (CK) and 4 °C treatments. The number of points and proportion represent the number of four different band types (A, B, C, and D) and their respective percentages to the total types. The pattern indicates changes in DNA methylation.

Type	CK		4 °C		CT-2360	MXW-1	2018-FJT	DT-7	Pattern
					Number of Points and Proportion	Number of Points and Proportion	Number of Points and Proportion	Number of Points and Proportion	
A (%)	EH	EM	EH	EM	114 (62.30%)	104 (55.62%)	110 (62.15%)	113 (58.55%)	no change
A1	1	1	1	1	71	64	67	56	
A2	1	0	1	0	19	16	14	25	
A3	0	1	0	1	24	24	29	32	
B (%)					27 (14.75%)	43 (22.99%)	35 (19.77%)	47 (24.35%)	demethylation
B1	1	0	1	1	7	6	2	4	
B2	0	1	1	1	7	14	10	16	
B3	0	0	0	1	3	11	6	13	
B4	0	0	1	0	6	8	14	8	
B5	0	0	1	1	4	4	3	6	
C (%)					42 (22.95%)	34 (18.18%)	27 (15.26%)	33 (17.10%)	methylation/ hypermethylation
C1	1	0	0	0	14	7	5	9	
C2	0	1	0	0	9	3	5	9	
C3	1	1	0	1	7	11	6	5	
C4	1	1	1	0	5	4	5	3	
C5	1	1	0	0	7	9	6	7	
D (%)					0 (0)	6 (3.21%)	5 (2.82%)	0 (0)	other
D1	1	0	0	1	0	2	4	0	
D2	0	1	1	0	0	4	1	0	
Total					183	187	177	193	

**Table 3:** Effects of room temperature recovery on the DNA methylation pattern in *Brassica rapa* L. The different types are shown in Figure 2. EH and EM represent the results of EcoRI/HpaII and EcoRI/MspI double digestion, respectively. The presence (1)/absence (0) were determined based on the polyacrylamide gel electropherograms of 18 polymorphic primer pairs during control (CK) and 24 °C recovery growth for 2 d (RG2d). The number of points and proportion represent the number of four different band types (A, B, C, and D) and their respective percentages to the total types. Pattern indicates changes in DNA methylation.

Type	CK		RG2d		CT-2360	MXW-1	2018-FJT	DT-7	Pattern
					Number of Points and Proportion	Number of Points and Proportion	Number of Points and Proportion	Number of Points and Proportion	
A (%)	EH	EM	EH	EM	77 (49.68%)	88 (53.99%)	86 (50%)	97 (48.99%)	no change
A1	1	1	1	1	41	52	49	47	
A2	1	0	1	0	13	15	15	26	
A3	0	1	0	1	23	21	22	24	
B (%)					21 (13.55%)	31 (19.02%)	38 (22.1%)	43 (21.72%)	demethylation
B1	1	0	1	1	8	7	2	6	
B2	0	1	1	1	3	5	10	9	
B3	0	0	0	1	5	5	9	9	
B4	0	0	1	0	3	11	10	11	
B5	0	0	1	1	2	3	7	8	
C (%)					56 (36.13%)	37 (22.7%)	45 (26.16%)	55 (27.78%)	methylation/ hypermethylation
C1	1	0	0	0	15	9	7	10	
C2	0	1	0	0	10	11	13	13	
C3	1	1	0	1	19	8	12	13	
C4	1	1	1	0	6	4	9	11	
C5	1	1	0	0	6	5	4	8	
D (%)					1 (0.64%)	7 (4.29%)	3 (1.74%)	3 (1.51%)	other
D1	1	0	0	1	0	3	2	3	
D2	0	1	1	0	1	4	1	0	
Total					155	163	172	198	

**Table 4:** Blast results of the differentially methylated DNA fragments in *Brassica rapa* L. Methylation occurs if the bands appear only in CK rather than the 4 °C treatment. However, demethylation takes place when the opposite is true. The homologs of *Brassica napus* and *Brassica rapa* genes are presented for the annotation of methylated fragments.

Fragment	Methylation Pattern	Length (bp)	Gene Description	E-Value	Identities %	Accession
M-01	Demethylation	167	*Brassica napus* protein KTI12 homolog (LOC106369112), transcript variant X2, mRNA	2.00 × 10^−46^	91.1	XM_013809215.2
M-02	Demethylation	143	*Brassica napus* NADH dehydrogenase [ubiquinone] 1 alpha subcomplex subunit 13-B (LOC106365400), mRNA	1.00 × 10^−53^	100	XM_013804832.2
M-03	Demethylation	75	*Brassica rapa* probable receptor-like protein kinase At5g39020 (LOC103874456), mRNA	3.00 × 10^−16^	100	XM_009152882.3
M-04	Methylation	128	*Brassica rapa* VQ motif-containing protein 22 (LOC103834848), mRNA	5.00 × 10^−42^	98.08	XM_009110941.3
M-05	Demethylation	142	*Brassica napus* cysteine-rich receptor-like protein kinase 10 (LOC106398281), mRNA	3.00 × 10^−49^	97.5	XM_013838867.2
M-06	Methylation	93	*Brassica rapa* cysteine-rich receptor-like protein kinase 10 (LOC103834757), transcript variant X4, mRNA	9.00 × 10^−28^	100	XM_033286881.1
M-07	Demethylation	170	*Brassica napus* glucan endo-1,3-beta-glucosidase (LOC106445206), mRNA	2.00 × 10^−66^	99.31	XM_013886724.2
M-08	Methylation	88	*Brassica rapa* glucan endo-1,3-beta-glucosidase (LOC103832330), transcript variant X2, mRNA	5.00 × 10^−20^	96.88	XM_009108313.3
M-09	Methylation	67	*Brassica rapa* glucan endo-1,3-beta-glucosidase 11 (LOC103835240), mRNA	1.00 × 10^−8^	95.35	XM_009111375.3
M-10	Demethylation	129	*Brassica rapa* putative glutamine amidotransferase GAT1_2.1 (LOC103850104), mRNA	9.00 × 10^−15^	98.08	XM_009126815.3
M-11	Methylation	133	*Brassica rapa* endoglucanase 8 (LOC103831605), mRNA	2.00 × 10^−25^	100	XM_009107492.3
M-12	Methylation	81	*Brassica rapa* O-glucosyltransferase rumi homolog (LOC103843476), mRNA	4.00 × 10^−15^	100	XM_009120210.3
M-13	Methylation	98	*Brassica rapa* auxin-induced protein 15A (LOC103834602), mRNA	3.00 × 10^−27^	98.65	XM_009110705.3
M-14	Demethylation	105	*Brassica rapa* transcription initiation factor IIB (LOC103863051), transcript variant X3, mRNA	3.00 × 10^−28^	95.35	XM_009140791.3
M-15	Demethylation	178	*Brassica rapa* terpenoid synthase 17 (LOC103859580), mRNA	4.00 × 10^−59^	94.81	XM_009137140.2
M-16	Methylation	71	*Brassica rapa* S-locus-specific glycoprotein S13 (LOC103831122), mRNA	8.00 × 10^−12^	97.83	XM_009106973.3
M-17	Methylation	64	*Brassica rapa* monothiol glutaredoxin-S1 (LOC103844538), mRNA	5.00 × 10^−8^	100	XM_033282469.1
M-18	Demethylation	58	*Brassica rapa* purple acid phosphatase 5 (LOC103837841), transcript variant X2, mRNA	5.00 × 10^−7^	97.3	XM_009114221.3
M-19	Demethylation	124	*Brassica rapa* iron-sulfur assembly protein IscA-like 1, mitochondrial (LOC117127666), mRNA	2.00 × 10^−41^	99	XM_033278271.1
M-20	Demethylation	124	*Brassica napus* homeobox-leucine zipper protein HDG2-like (LOC106398145), mRNA	2.00 × 10^−41^	99	XM_013838740.1
M-21	Methylation	109	*Brassica rapa* elongation factor G-2, mitochondrial (LOC103866857), mRNA	5.00 × 10^−31^	97.62	XM_009144847.3
M-22	Methylation	97	*Brassica rapa* probable protein phosphatase 2C 30 (LOC103865774), mRNA	3.00 × 10^−28^	100	XM_009143626.2
M-23	Methylation	184	*Brassica rapa* probable clathrin assembly protein At4g32285 (LOC103851149), mRNA	2.00 × 10^−77^	100	XM_009127985.3
M-24	Demethylation	74	*Brassica rapa* E3 ubiquitin-protein ligase ATL6-like (LOC103859080), mRNA	1.00 × 10^−5^	100	XM_009136564.3
M-25	Demethylation	75	*Brassica rapa* testis-expressed protein 2 (LOC103831800), mRNA	1.00 × 10^−5^	97.14	XM_009107719.3
M-26	Methylation	89	*Brassica rapa* glycine-rich RNA-binding protein 5, mitochondrial-like (LOC103860185), mRNA	3.00 × 10^−22^	98.46	XM_033288947.1
M-27	Methylation	113	*Brassica napus* polyribonucleotide nucleotidyltransferase 2, mitochondrial-like (LOC106416529), transcript variant X3, mRNA	3.00 × 10^−14^	98.04	XM_013857446.2
M-28	Demethylation	91	*Brassica napus* protein DETOXIFICATION 37 (LOC106419146), transcript variant X2, mRNA	1.00 × 10^−25^	96.15	XM_013859997.2
M-29	Demethylation	93	Cardamine resedifolia voucher CresL-Sta1-1 haplotype 1 Ascorbate peroxidase 1 (APX1) gene, partial cds	4.00 × 10^−11^	95.92	KJ428054.1
M-30	Demethylation	75	*Brassica rapa* subsp. pekinensis cultivar Inbred line Chiifu “clone KBrB045B23, complete sequence”	3.00 × 10^−16^	96.49	AC189360.2
M-31	Demethylation	164	*Brassica juncea* isolate 94 mitochondrion, complete genome	1.00 × 10^−48^	92.86	MG872828.1
M-32	Demethylation	164	*Brassica juncea* cytoplasmic male sterility-associated cytochrome oxidase subunit I (coxI-2) gene, complete cds; mitochondrial gene for mitochondrial product	7.00 × 10^−52^	94.29	AY300015.1
M-33	Methylation	75	Pseudomonas fluorescens strain NCTC10038 genome assembly, chromosome: 1	1.00 × 10^−14^	96.3	LS483372.1
M-34	Methylation	179	*Brassica oleracea* HDEM genome, scaffold: C4	3.00 × 10^−65^	96.77	LR031873.1
M-35	Methylation	102	*Tetracme recurvata* isolate C236 18S ribosomal RNA gene, internal transcribed spacer 1, 5.8S ribosomal RNA gene, internal transcribed spacer 2, and 26S ribosomal RNA gene, complete sequence	6.00 × 10^−30^	97.56	MT819350.1
M-36	Methylation	214	*Brassica juncea* chromosome og1-b mitochondrion, complete sequence	2.00 × 10^−92^	100	MT675106.1
M-37	Demethylation	104	Uncultured bacterium clone Otu03067 16S ribosomal RNA gene, partial sequence	2.00 × 10^−29^	97.53	KX997786.1
M-38	Methylation	155	*Yosemitea repanda* chloroplast, complete genome	6.00 × 10^−47^	100	MK637830.1
M-39	Methylation	128	*Lepidium apetalum* chloroplast, complete genome	4.00 × 10^−18^	100	MT880914.1
M-40	Methylation	81	*Brassica rapa* uncharacterized LOC103836772, transcript variant X2, mRNA	1.00 × 10^−6^	93.18	XM_033280897.1
M-41	Demethylation	154	*Brassica rapa* uncharacterized LOC103871127, mRNA	1.00 × 10^−9^	100	XM_009149356.3
M-42	Demethylation	89	*Brassica rapa* uncharacterized LOC103874467, mRNA	5 × 10^−20^	96.88	XM_009152895.3
M-43	Demethylation	109	*Brassica rapa* uncharacterized LOC103833097, transcript variant X4, misc_RNA	7.00 × 10^−35^	98.86	XR_626125.2
M-44	Methylation	68	*Brassica rapa* uncharacterized LOC117128160, ncRNA	0.002	100	XR_004451357.1
M-45	Demethylation	97	*Brassica rapa* uncharacterized LOC117134590, ncRNA	3.00 × 10^−18^	100	XR_004458779.1
M-46	Demethylation	92	*Brassica rapa* uncharacterized LOC117127533, mRNA	4.00 × 10^−26^	100	XM_033278112.1
M-47	Demethylation	87	*Brassica rapa* uncharacterized LOC103874342, transcript variant X2, mRNA	8.00 × 10^−23^	95.89	XM_033292971.1
M-48	Methylation	75	*Brassica rapa* uncharacterized LOC103846332, mRNA	1.00 × 10^−26^	97.37	XM_009123242.3
M-49	Demethylation	53	*Brassica napus* uncharacterized LOC106386228, mRNA	0.001	100	XM_013826104.2
M-50	Methylation	108	*Brassica napus* uncharacterized LOC106378351, mRNA	5.00 × 10^−31^	98.77	XM_013818495.1
M-51	Methylation	87	*Brassica napus* uncharacterized LOC106388033, transcript variant X2, ncRNA	2.00 × 10^−14^	92.06	XR_001277777.2
M-52	Methylation	188	*Brassica napus* uncharacterized LOC106451272, mRNA	1.00 × 10^−79^	98.3	XM_013893176.2
M-53	Demethylation	121	*Brassica napus* uncharacterized mitochondrial protein LOC111207234, mRNA	1.00 × 10^−13^	100	XM_022705092.1

**Table 5:** Geographic and climatic factors, cold resistance indicators, and agronomic traits of the four tested materials. Root collar diameter, overwintering rate, LT50, and cold resistance ranking are cold resistance indicators, and plant height, branch height, siliques per plant, seeds per silique, thousand-seed weight, and seed yield per plant are key agronomic traits.

Materials	Place of Origin and Environmental Conditions	Root Collar Diameter (cm)	Over- Wintering Rate (%)	LT50 (°C)	Cold Resistance Ranking	Plant Height (cm)	Branch Height (cm)	Siliques per Plant	Seeds per Silique	Thousand-Seed Weight (g)	Seed Yield per Plant (g)
CT-2360	Caotan, Zhangxian County, Gansu Province; Latitude: 34°38′ N; longitude: 104°28′ E; above sea level: 2360~2460 m; mean annual temperature: 4.3 °C; lowest annual temperature: −8.8 °C.	1.1	89.0	−11.32	4	112.0	11.2	145.0	21.5	3.1	11.0
MXW-1 (Longyou-7)	Shangchuan, Lanzhou City, Gansu Province; latitude: 36°03′; longitude: 103°40′; above sea level: 2150 m; mean annual temperature: 6.5 °C; lowest annual temperature: −14.6 °C.	1.0	90.0	−15.04	3	102.0	8.2	150.0	22.0	3.1	12.0
DT-7	Datong City, Shanxi Province; Latitude: 40°04′; longitude: 113°08′; above sea level: 1000 m; mean annual temperature: 6.4 °C; lowest annual temperature: −29.2 °C.	1.0	94.0	−15.98	2	92.0	8.0	157.0	22.0	3.2	12.5
2018-FJT	Fanjiatun, Gongzhuling City, Jilin Province; Latitude: 43°43′; longitude: 124°50′; above sea level: 223 m; mean annual temperature: 5.9 °C; lowest annual temperature: −30 °C.	1.1	95.0	−16.04	1	90.0	6.1	167.0	23.0	3.3	13.2

## Data Availability

The data presented in this study are available in the article and Supplementary Materials.

## Supplementary Materials

The following supporting information can be downloaded at: https://www.mdpi.com/article/10.3390/plants13131748/s1. Figure S1: Electrophoretic diagram of pre-amplified products; Table S1: Sequence of adapters and primers used in MSAP technology; Table S2: Selected polymorphic primers; Table S3: Primer sequence.
