# Supplementary material for: Methyl-Sensitive Amplification Polymorphism (MSAP) Analysis Provides Insights into the DNA Methylation Changes Underlying Adaptation to Low Temperature of Brassica rapa L
Source: Plants (Basel). 2024 Jun 24;13(13):1748. doi: 10.3390/plants13131748 (PMC11244143; doi:10.3390/plants13131748)
Supplement: Supplementary file 1 [file plants-13-01748-s001.zip › plants-3014536-supplementary.pdf]

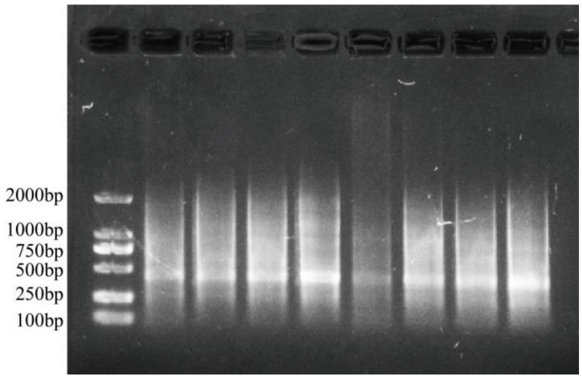

Figure S1 Electrophoretic diagram of pre-amplified products

Table S1 Sequence of adapters and primers used in MSAP technology

	EcoRI (5'-3')	Sequences	HpaII/MspI (5'-3')	Sequences
adapter	adapter	5'-CTCGTAGACTGCGTACC-3'<br>5'-AATTGGTACGCAGTCTAC-3'	adapter	5'-GACGATGAGTCTAGAA-3<br>5'-CGTTCTAGACTCATC-3'
pre-amplified primers	E-pre	5'-GACTGCGTACCAATTCA-3'	HM-pre	5'-GATGAGTCTAGAACGGT3'
selected primers	1	GACTGCGTACCAATTCATA	32	GATGAGTCTAGAACGGTAG
	4	GACTGCGTACCAATTCAGA	33	GATGAGTCTAGAACGGTAC
	6	GACTGCGTACCAATTCAGT	37	GATGAGTCTAGAACGGTGG
	10	GACTGCGTACCAATTCACC	312	GATGAGTCTAGAACGGTCC
	15	GACTGCGTACCAATTCAAT	313	GATGAGTCTAGAACGGTTA
	37	GACTGCGTACCAATTACG	315	GATGAGTCTAGAACGGTTC
	47	GACTGCGTACCAATTCAA		
	50	GACTGCGTACCAATTCAT		
	75	GACTGCGTACCAATTCGTA		
	86	GACTGCGTACCAATTCTCT		

Table S2 Selected polymorphic primers

Number	Primer combination	Number	Primer combination	Number	Primer combination
1	E4/HM32	7	E86/HM37	13	E4/HM313
2	E6/HM32	8	E1/HM312	14	E37/HM313
3	E37/HM32	9	E6/HM312	15	E86/HM313
4	E86/HM32	10	E10/HM312	16	E1/HM315
5	E6/HM37	11	E37/HM312	17	E10/HM315
6	E37/HM37	12	E1/HM313	18	E15/HM315

Table S3 Primers sequence

Gene	Primer	Sequence(5'to3')
VQ22 (clone)	FP	ATGGCTAACAACCCAAACGA
	RP	TCATTGCAGCCTCGAAGACG
LOC103871127 (clone)	FP	ATGTCTGTGGAGAGACCCTT
	RP	TCAGAACTCAAAAGCATTGT
β-actin (reference gene of qPCR)	FP	GTGTCATGGTTGGGATGGGT
	RP	AAGAACCGGGTGCTCTTCAG
VQ22 (qPCR)	Con-FP	AGCCTCACGTAGAACACCAACAAC
	Con-RP	GATGGACCGCCCGTGTATTGC
	Spe-FP	CTCCACCGCAACGGCCTTATATG
	Spe-RP	AGAACCTCCTGCTCCACCATCC
LOC103871127 (qPCR)	Con-FP	GCCATCAGAAACTGAAGCTGTTCG
	Con-RP	CCTCCACGCCGCCATTGATG
	Spe-FP	AGATGCTGTCTCCTCTGCTGT
	Spe-RP	ACCGTTCCCTTTGGTCTCCT

Note: FP, Forward primer; RP, Reverse primer; Con: Conservative sequence; Spe: Specific sequence.
